# Anomalous left coronary artery from the pulmonary artery presenting with aborted sudden death in an octogenarian: a case report

**DOI:** 10.1186/1752-1947-6-12

**Published:** 2012-01-16

**Authors:** Ahmad Separham, Parvaneh Aliakbarzadeh

**Affiliations:** 1 Department of Cardiology, Cardiovascular Research Center, Shahid Madani Heart Hospital, Tabriz, Iran

## Abstract

**Introduction:**

We report a rare coronary anomaly presenting with aborted sudden death in an octogenarian. An anomalous left coronary artery from the pulmonary artery is a rare coronary anomaly which usually presents in the first year of life. Survival into adulthood and the elderly years is extremely rare.

**Case presentation:**

An 85-year-old Caucasian woman was brought to our hospital following cardiopulmonary arrest. After prolonged resuscitation and stabilization of our patient, further evaluation revealed an anomalous left coronary artery from pulmonary artery syndrome. She was discharged on medication.

**Conclusion:**

An anomalous left coronary artery from the pulmonary artery can present in elderly and even octogenarian patients. Careful history, physical examination and an appropriate invasive study are needed to confirm the diagnosis.

## Introduction

Anomalous origin of the left main coronary artery from the pulmonary artery (ALCAPA), also known as Bland-White-Garland syndrome is a rare congenital anomaly occurring in one in 300,000 births [1].The typical clinical course is severe left-sided heart failure presenting at the age of one to two months [2]. Without surgical intervention, most patients with ALCAPA die within the first year of life [3]. In adult life, symptoms may range from dyspnea, chest pain and exercise intolerance to sudden cardiac death. We describe a case of an ALCAPA in an 85-year-old woman who presented with aborted sudden death.

### Case presentation

**Figure 1:**
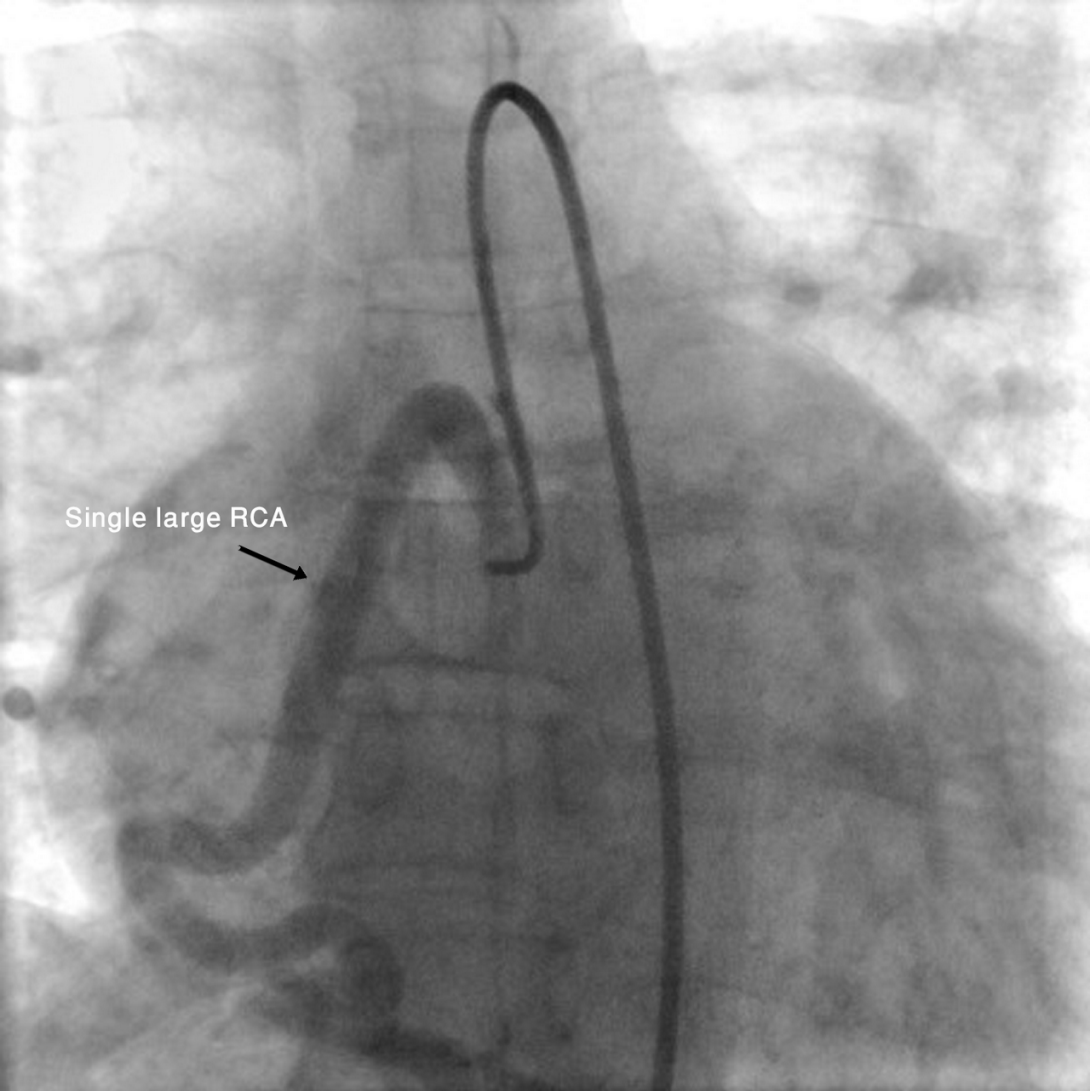
**Selective right coronary angiography shows a widely dilated right coronary artery**.

**Figure 2:**
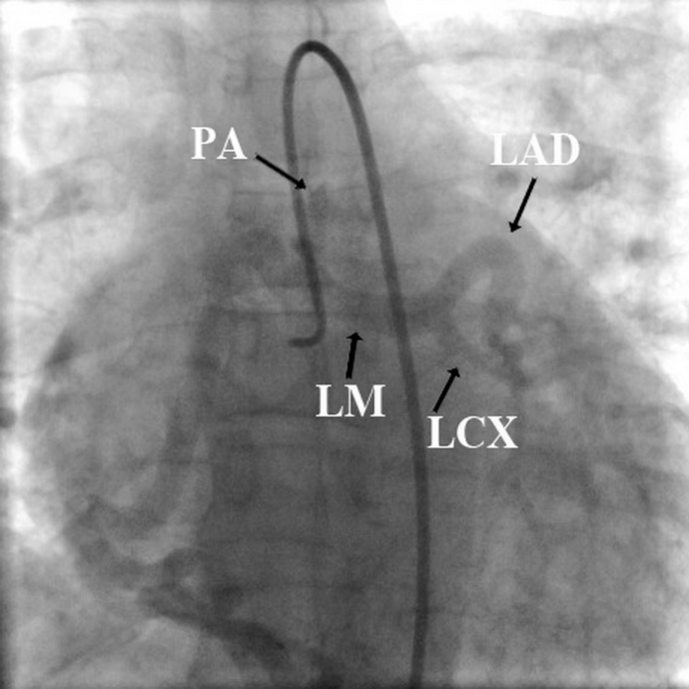
**Retrograde filling of the left anterior descending artery, left circumflex artery and main pulmonary artery on delayed imaging after contrast injection of the right coronary artery**.

An 85-year-old Caucasian woman experienced sudden loss of consciousness during walking. She was brought to our hospital and found to be in ventricular fibrillation. After prolonged resuscitation, our patient converted to sinus rhythm with stable hemodynamics. She had no coronary risk factors or history of cardiovascular disease. A cerebral computed tomography scan was found to be normal. Her serial cardiac enzymes were negative and an electrocardiogram had non-specific ST-T changes. Echocardiography showed reduced left ventricle systolic performance with an ejection fraction of 40%, global hypokinesia and mild mitral regurgitation. There was no evidence of her previous echo findings or left ventricular ejection fraction. After three days and extubation of our patient, a coronary angiography and cardiac catheterization was performed. Her left main coronary artery could not be selectively engaged. A selective right coronary injection using a right Judkins catheter showed a large and tortuous right coronary artery arising from the right sinus of Valsalva. The left coronary artery was filled through collaterals from the right coronary artery (RCA). The anomalous origin of the left coronary artery was demonstrated in the late phase of RCA injection (Figures 1 and 2, Additional file 1). The calculated left to right shunt (pulmonic blood flow to systemic blood flow ratio) was 1.01. A diagnosis of ALCAPA syndrome was confirmed. She was discharged home with stable cardiovascular and neurologic status a few days later.

## Discussion

ALCAPA is a rare congenital anomaly, first described by Brooks in 1886. It is usually seen as an isolated lesion. This anomaly, also known as Bland-White-Garland syndrome, accounts for about 0.25% to 0.5% of congenital heart defects [4]. Symptoms usually occur in the first few months of life. Late presentation in the adult or elderly stage of life is extremely rare. Insufficient collateral flow from the right coronary artery with a coronary steal from the left coronary artery into the pulmonary trunk result in malperfusion of the left ventricular myocardium, with the right coronary artery becoming large and tortuous. Previous existence or the rapid development of collateral vessels between the right and the left coronary arteries may prevent ischemia. To the best of our knowledge, our patient is one of the oldest reported cases in the literature. Our patient's lack of symptoms until presentation may be related to extensive collateral vessels between the left and right coronary arteries, which provided enough oxygenated blood to her myocardium. But, the cause-effect relationship between ALCAPA and ventricular fibrillation in this elderly patient cannot be definitely proved.

Surgery is considered the treatment of choice for this anomaly. Various surgical methods have been attempted, including simple ligation, bypass grafts and reimplantation of coronary arteries in the aorta [5]. Considering our patient's old age and her family request she was discharged home on medication including acetylsalicylic acid, amiodarone and losartan.

## Conclusion

In this article we describe an elderly woman presenting with ventricular fibrillation, who was found to have a coronary anomaly. To the best of our knowledge the patient in this case is one of the oldest, or may be the oldest, patient with this type of coronary anomaly, known as ALCAPA syndrome, who survived up to 85 years of age. It behooves one to consider coronary anomaly as a possible cause of sudden death even in an octogenarian.

## Consent

Written informed consent was obtained from the patient for publication of this case report and any accompanying images. A copy of the written consent is available for review by the Editor-in-Chief of this journal.

## Competing interests

The authors declare that they have no competing interests.

## Authors' contributions

PA resuscitated our patient and stabilized our patient's clinical status. AS performed the coronary angiography and confirmed the diagnosis. Both authors read and approved the final manuscript.

## Supplementary Material

Additional file 1 **Selective right coronary angiography at anteroposterior (AP) view reveals a single large right coronary artery with retrograde filling of the left anterior descending artery (LAD), left circumflex artery (LCX), Left Main (LM) and pulmonary artery(PA)**. Click here for file
